# Sexual Orientation and Gender Identity Change Efforts for Young People in New Zealand: Demographics, Types of Suggesters, and Associations with Mental Health

**DOI:** 10.1007/s10964-022-01693-3

**Published:** 2022-10-27

**Authors:** John Fenaughty, Kyle Tan, Alex Ker, Jaimie Veale, Peter Saxton, Mohamed Alansari

**Affiliations:** 1 School of Counselling, Human Services and Social Work, Faculty of Education and Social Work, University of Auckland, Private Bag 92019, Auckland, 1142 New Zealand; 2 Faculty of Māori and Indigenous Studies, University of Waikato, Private Bag 3105, Hamilton, 3240 New Zealand; 3 Trans Health Research Lab, School of Psychology, University of Waikato, Private Bag 3105, Hamilton, 3240 New Zealand; 4 School of Population Health, Faculty of Medical and Health Sciences, University of Auckland, Private Bag 92019, Auckland, 1142 New Zealand; 5 New Zealand Council for Educational Research, 10 Brandon Street, Wellington, 6011 New Zealand

**Keywords:** Conversion therapy, SOGICE, Transgender, LGBT, NSSI, Suicidality

## Abstract

Sexual orientation and gender identity change efforts (SOGICE) are harmful practices, yet who suggests them to young people and what impacts are associated with these suggestions have received limited attention in the literature. The present study explored whether certain suggesters, and the frequency of categories of suggesters (including religious leaders, family members, and health professionals), were associated with suicidality and non-suicidal self-injury (NSSI). The study also explored whether particular demographics of young people were more likely to report SOGICE experiences. Data were collected through an online survey of New Zealand gender- and sexuality-diverse youth. The sample (*n* = 3948) had an age range of 14–26 (mean age = 18.96), and approximately half (52.4%) were transgender or gender-diverse. Odds of suicidality and NSSI were highest when religious leaders suggested SOGICE and when more than one type of suggester was reported. SOGICE was more likely to be reported by transgender and gender-diverse youth, statutory care- and homelessness-experienced youth, and young people reporting current material deprivation. Implications for targeted mental health services and education for young people and the community are discussed.

## Introduction

Sexual orientation and gender identity change efforts (SOGICE) are pseudoscientific practices that intend to alter diverse gender (American Psychological Association, 2021a) and sexual (American Psychological Association, 2021b) identities. Serious mental health outcomes, including suicidality (Blosnich et al., 2020) and non-suicidal self-injury (NSSI) (Veale et al., 2021), have been associated with SOGICE. Although most studies on SOGICE have primarily focused on adults, the impacts of SOGICE during adolescence are important to consider. Adolescence and young adulthood have been characterized as periods of intense identity development (Waterman, 1982). To the extent that SOGICE disrupts positive identity development, it also threatens gender- and sexuality-diverse young people’s wellbeing. Young people develop in a range of contexts, including families, schools and religious communities. These are important contexts where suggestions of SOGICE may have significant impacts on young people’s development and wellbeing. This study explored the prevalence, demographics, and effects of SOGICE in a youth-specific survey and identified who suggested SOGICE to young people and whether the particular categories of people and the frequencies of types of people, who suggested SOGICE were associated with harm.

The wellbeing of gender- and sexuality-diverse young people^1^ is a pressing issue. Meta-analyses have consistently demonstrated a higher prevalence of non-suicidal self-injury (NSSI) and suicidality for gender- and sexuality-diverse young people compared to cisgender heterosexual young people in many nations (Plöderl & Tremblay, 2015). Mental health disparities have also been identified for transgender and gender-diverse (Fenaughty et al., 2021a) and sexuality-diverse youth (Fenaughty et al., 2021b) in New Zealand. The elevated risks of NSSI and suicidality for young people in New Zealand and internationally occur in the context of cisheterosexist stigma and discrimination against people with transgender and gender-diverse (Tan et al., 2022) and sexuality-diverse (De Lange et al., 2022) identities. SOGICE represents a particularly acute aspect of this damaging cisheterosexism.

### Conceptualizing SOGICE

Cisheterosexism is a system of beliefs and practices that center heterosexual cisgender identities as the norm (Cowie & Braun, 2021). Cisheterosexism is produced and sustained by heteronormativity. Heteronormativity, the system that constructs heterosexuality as the only normal, natural and desirable sexuality, is enabled by the cissexist assumption that gender only exists as a woman/man binary. Heteronormativity operates through a “heterosexual matrix” that requires “…a stable sex expressed through a stable gender (masculine expresses male, feminine expresses female) that is oppositionally and hierarchically defined through the compulsory practice of heterosexuality” (Butler, 1990, p.151). Departures from heteronormative alignment in this matrix (i.e., male assigned sex, masculine gender, and heterosexual attraction to women; female assigned sex, feminine gender, and heterosexual attraction to men) place people outside of the heteronorm, where they may be subjected to stigma and sanctions, including SOGICE. Although sex, gender, and sexuality are distinct concepts, the pervasiveness of heteronormativity, through the action of the heterosexual matrix, often leads to the conflation of all three. For example, US research (Pascoe, 2005) shows how homophobic language and “fag discourse” (p.330) can be used to police gender non-conformity by conflating feminine male behaviour with stereotypes of gay males. To date, the research on SOGICE varies in whether it assesses practices targeting sexual orientation and gender identity distinctly or collectively; however, the American Psychological Association (2021b) notes that these practices are not always mutually exclusive.

Practices of SOGICE manifest in various forms, including structured programs at formal institutions (e.g., a course at a church), medication (Chan et al., 2022) or medical interventions (e.g., electroshock and aversion therapy), and psychotherapeutic techniques, including counselling (Salway et al., 2021). SOGICE also includes attempting to change or suppress sexual orientation or gender identity in healthcare settings, including withholding access to gender-affirming healthcare (Veale et al., 2021). Although certain SOGICE practices may operate more insidiously (e.g., ‘theological’ conversations with a religious leader or group prayer meetings to ‘help/save’ an absent/excluded individual), they all involve pervasive efforts to reinforce cisgender and heterosexual norms (Salway et al., 2021).

### Prevalence of SOGICE

To date, studies with majority-adult populations from nonprobability samples demonstrate lifetime estimates of sexual orientation change efforts ranging from 3% in Korea (*n* = 2168; Lee et al., 2021) to 22% in Hong Kong (*n* = 219; Chan et al., 2022), and gender identity conversion efforts ranged from 14% in the United States (*n* = 27,676; Turban et al., 2019) to 20% in Aotearoa New Zealand (*n* = 610; Veale et al., 2021). A Canadian study explored the prevalence of conversion therapy practices in 9214 sexual minority men in Canada (Salway et al., 2021). In the Canadian sample, 9.9% (*n* = 910) of participants reported experiencing conversion efforts from a “licensed healthcare professional (psychologist, psychiatrist, doctor), unlicensed counselor, camp, faith-based organization focused on conversion therapy, individual religious leader (i.e., not through a formal organization), and/or another religious individual” (Salway et al., 2021, p.4). However, when asked a broader question about SOGICE exposure, “have you or any person with authority (parent, caregiver, counselor, community leader, etc.) ever tried to change your sexual orientation or gender identity?” (Salway et al., p. 4), the proportion of participants reporting lifetime SOGICE experiences doubled to 20.6%.

There are a few potential explanations for the differences in the prevalence of SOGICE across these studies, including the age distribution of a sample, the range of measures used to identify SOGICE and whether they include subtler forms of SOGICE, as well as the cultural and political contexts of each country. Younger adults have reported a higher prevalence of change efforts than adults and older adults in studies in the Southern USA (Higbee et al., 2020) and Canada (Salway et al., 2021). In New Zealand, transgender adolescents aged 14–18 years were significantly more likely to be exposed to gender identity change efforts than older adults aged ≥55 years (25 vs 15%) (Veale et al., 2021). However, limited work has focused solely on the prevalence of SOGICE in children and young adults. To date, one large nonprobability sample (*n* = 25,791) from the USA found that 4.2% of gender- and sexuality-diverse youth and young adults reported experiencing SOGICE in their lifetime (Green et al., 2020). In that youth-specific study, sexual orientation change efforts were not assessed separately from gender identity change efforts and participants aged 13–17 years were more likely to report SOGICE than those aged 18–24 (5.1 vs 3.3%).

A broad range of people initiate SOGICE, including leaders in religious and spiritual groups, family members and relatives, and health care providers. Canadian research found that approximately two-thirds of those who reported SOGICE said it was initiated by religious leaders or individuals and faith-based organizations; the next common initiators were licensed health professionals; and one-fifth reported SOGICE by unlicensed health professionals (Salway et al., 2021). New Zealand research of transgender and gender-diverse people’s experiences of gender identity change efforts found that SOGICE has also been initiated by mental health professionals, including psychiatrists, psychologists, and counsellors (Veale et al., 2021).

Transgender and gender-diverse participants in New Zealand research who were exposed to SOGICE in health care settings were more likely to report having been rejected by their family members for being transgender (Veale et al., 2021). Indeed, other research has found that individuals themselves may initiate SOGICE because they fear being rejected by their family and religious communities for being gender or sexuality-diverse (Chan et al., 2022). Hong Kong research found that the desire to be heterosexual and avoid gender- and sexuality-diverse stigma were strong drivers for sexuality-diverse men to undertake sexual orientation change efforts (Chan et al., 2022). Studies involving sexual- (Chan et al., 2022) and gender- (Veale et al., 2021) diverse participants have shown higher levels of internalized homo/bi/transphobia among those exposed to SOGICE.

### Mental Health Harms Associated with SOGICE

Not only do SOGICE lack an adequate basis in scientific and ethical principles (Przeworski Peterson & Piedra, 2021), they are, in fact, associated with a range of significant mental health issues that are most likely produced by the stress, trauma and harm caused by these practices. International literature indicates an association between exposure to SOGICE and detrimental mental health outcomes among predominantly adult samples, including anxiety symptoms (Chan et al., 2022), depression symptoms (Lee et al., 2022), suicidal thoughts (Ryan et al., 2020); and suicide attempts (Turban et al., 2020). The psychological impact of SOGICE may be more acute for young people, given the developmental tasks of identity development and consolidation that are most salient at this age (Waterman, 1982). In a youth-specific study in the USA (Green et al., 2020), those who reported SOGICE had about twice the odds of seriously considering suicide (63 vs 38%; odds ratio [OR] 1.76) and attempting suicide (44 vs 18%; OR 2.23) than those without such experiences.

### Current Legislation Restricting SOGICE

As of 2022, five countries—Brazil, Malta, Germany, Canada (Mendos, 2020) and Ecuador (Horne & McGinley, 2022)—have implemented national laws explicitly restricting SOGICE, though the scopes of the respective legislation vary. For example, Brazil’s laws only restrict licensed psychologists from practicing SOGICE; in contrast, the legislation in Malta prevents both non-professionals and professionals from practicing SOGICE (Mendos, 2020), and though Germany’s laws only protect minors, it includes family members within its scope of people who may be held accountable by the law (BBC, 2020). Some countries have regional prohibitions, including Australia, the United States, and Spain. In contrast, several countries in Latin America and Oceania have laws that indirectly pose restrictions on forms of SOGICE (such as prohibiting a mental health professional from diagnosing someone as mentally ill based on their gender or sexuality) but do not explicitly name or define conversion practices in such laws (Mendos, 2020). In February 2022, Aotearoa New Zealand’s government voted to pass the Conversion Practices Prohibition Legislation Bill into law. Several other countries are considering passing legislation to prohibit these practices.

Human rights instruments are frequently used to sanction laws against SOGICE (Mendos, 2020). Critical tensions revolve around gender- and sexuality-diverse people’s freedom of expression and freedom from torture versus proponents’ freedom of religion and freedom of parental rights. Proponents of SOGICE, including some political leaders, claim religious leaders’ and parents’ rights to promote and conduct SOGICE outweigh the need to act in the best interests of the child (Mendos, 2020). However, the United Nations Convention on the Rights of the Child (UNCRC), relevant to young people aged up to 18 years, states that the maturing young person is particularly vulnerable to adversity and “needs special safeguards and care, including appropriate legal protection” (p. 1). The UNCRC emphasizes that decisions around rights and protected practices must center on ensuring that the best interests of the child (as opposed to those of parents or religious leaders) are met. The UNCRC adds a buttress to gender- and sexuality-diverse young people’s rights to freedom of expression of their gender and sexuality identities, freedom from experiences of SOGICE-related torture (Méndez, 2013), and their right to life (a particular issue related to the suicide ideation associated with SOGICE).

## Current Study

As most of the studies on SOGICE to date have analyzed adult populations, there is limited understanding of young people’s contemporary experiences of SOGICE. This is the first youth-specific study outside the North American region to examine SOGICE experiences. The study aims to identify whether particular groups within a gender- and sexuality-diverse youth sample in Aotearoa New Zealand are more likely to report SOGICE and whether SOGICE is associated with NSSI, suicidal thoughts, suicide planning, and suicide attempts in this age group. The study also aims to examine whether there are differences in mental health outcomes for those exposed to SOGICE-suggestion by family members, religious leaders, medical professionals, and the young people themselves. Based on the literature, young people exposed to SOGICE are predicted to be more likely to report NSSI and suicidality. There has been no prior research examining whether the relationship between SOGICE and mental health differs by SOGICE suggestions from family, religious, medical, or self-suggested SOGICE in young people, so this exploratory study is not able to make informed predictions about whether any categories, or frequencies, of SOGICE-suggesters will be more strongly associated with NSSI and suicidality.

## Method

### Design and Consultation

Data was used from the Identify Survey, which was open from February to August 2021. Identify was an anonymous online survey available to takatāpui,^2^ MVPFAFF+,^3^ LGBTQIA+ young people, and friends and allies aged between 14 and 26 years (inclusive) in Aotearoa New Zealand. The survey focused on young people’s experiences across various contexts, including education, employment, home, and the community. The survey included questions on protective aspects and challenges in these contexts. A section also collected health and wellbeing data, including measures of suicide ideation and attempts.

The survey was a collaboration between two national youth community organizations and researchers who represented a range of genders, sexualities, ethnicities, and ages. The survey content, structure, recruitment, and branding were informed by nine in-person regional community consultations in 2020. Questions in this study were either developed by the research team, often following community consultation, or were replicated from existing New Zealand studies with transgender and gender-diverse people (Veale et al., 2021) and a national youth behavioral surveillance study (Fleming et al., 2020).

The survey was constructed in Qualtrics and supported smart logic so that participants were only shown questions relevant to their previous answers. In-person recruitment was conducted at community events, including Pride festival events in main cities and existing nightclub events and community meetings. Posters were placed in prominent community venues (e.g., queer- and trans-friendly bars and cafes), schools and tertiary institutions, and in the libraries of two large cities. Online recruitment was conducted via advertisements and posts on Facebook, Instagram, TikTok, Twitter, YouTube, and Grindr. Word of mouth, including via social media and preliminary data “teasers” in mainstream media stories, also advertised the survey. The study received ethical approval from the New Zealand Health and Disability Ethics Committee (20/NTB/276).

### Participants

The survey received 6712 initial responses. After filtering responses that were flagged by Qualtrics as spam (*n* = 86) or that did not provide consent (*n* = 39), did not meet age requirements (*n* = 511), were not living in Aotearoa New Zealand (*n* = 33), were duplicates (*n* = 35), were illogical, including homophobic and transphobic responses (*n* = 19), or did not complete more than five questions after the branching question on current educational or employment status (*n* = 748), the sample consisted of 5241 valid responses. The completion rate for the SOGICE section was high (*n* = 4118; 78.6%). In this study, participants were warned about a range of sensitive topics before sensitive sections and were given the opportunity to easily skip each section by selecting the option: “*I find this topic upsetting, and would like to skip these questions*.” Of the 4118 participants who began the section on SOGICE, some responded that they found the topic upsetting and wanted to skip the section (*n* = 129) and 41 selected “*I’d prefer not to say*” when asked the first question of this section about whether they had experienced conversion therapy; these participants (see Appendix Table 5) were excluded from the statistical analysis, leaving a final sample size of 3948.

The mean age of the sample was 18.96 (range 14–26; standard deviation [SD] 3.65). About half identified as cisgender (47.6%), and others included nonbinary (16.9%), unsure if trans/questioning (13.2%), trans boy/man (9.7%), another gender (8.1%), and trans girl/woman (4.4%). Based on the concept of total response (i.e., participants can be counted as more than one ethnic group) (Ministry of Health, 2017), a majority of participants identified as New Zealand European/Pākehā^4^ ethnicity (84.2%), followed by 14.5% indigenous Māori, 4.1% Chinese, 2.7% Indian, 2.5% British, 1.9% Filipino, 1.9% Samoan, 1.4% Dutch, and 1.0% Cook Island Māori. Participants were also able to select multiple responses to describe their sexuality. About half were queer (46.0%), and other identities were 43.4% bisexual, 23.5% gay, 23.4% pansexual, 19.6% lesbian, 14.8% fluid, 14.5% asexual, 9.5% demisexual, 7.8% unsure/questioning, and 5.9% takatāpui.

### Measures

#### Sociodemographics

Participants’ gender was determined by responses to two questions on gender and sex assigned at birth. Participants were asked, “When a person’s gender is different from their sex assigned at birth, they might think of themselves as transgender (or trans). Which of these statements best describe you? (Please select all that apply).” These statements were, *I am not [emphasis in original] transgender or nonbinary* (cisgender); *I am transgender and identity as a girl/woman/wahine* (trans girl); *I am transgender and identity as a boy/man/tāne* (trans boy); *I am transgender and identify with another gender* (another gender); *I am nonbinary* (nonbinary); *I’m not sure if I am transgender or nonbinary* (unsure). In this study, participants were assigned into one of the six categories, following the priority order of unsure, trans girl (assigned male at birth)/trans boy (assigned female at birth), another gender, nonbinary, and cisgender. For example, a participant who selected both “unsure” and “another gender” was categorized as having an “unsure” gender.

The sexuality of participants was collected with a question that asked, “Which of the following bestdescribe your sexuality? (Please select all that apply).” Response options were *Takatāpui; Queer; Gay; Lesbian; Bisexual; Pansexual; Fa'afafine; Fakaleiti; Heterosexual /straight; Mostly straight; Asexual; Aromantic; Demisexual; Fluid / it changes; Something else (please describe);* and *I’m not sure*.

**Table 1:** Sample proportion reporting SOGICE, categories of suggesters, and frequencies of types of suggesters (*N* = 4118)

Variable	*n*	%
SOGICE reported		
Yes	124	3.0
No	3824	92.9
“Find this topic upsetting and would like to skip OR I’d prefer not to say”	170	4.1
Categories of people who suggested SOGICE (*n* = 124)		
Family/whānau member	69	55.6
Leader in religious or spiritual community	60	48.4
Myself	22	17.7
Another person	18	14.5
Medical professional	14	11.3
Frequency of SOGICE suggester types^a^ (*n* = 3948)		
0	3834	97.1
1	70	1.8
2	41	1.0
3	3	~0.1

^a^Self-suggested SOGICE was excluded

A Canadian material deprivation index developed by the McCreary Centre (Smith et al., 2019), with local adaptations, was used to assess deprivation. The index provided a list of resources that are material wellbeing indicators for young people: *money for myself*; *smartphone*; *space to hang out on my own*; *money to spend on eating out*; a*ccess to transport*; *equipment or clothes for extracurricular activities*; *clothes that fit me*; *a quiet place to sleep*; and *access to high-quality internet*. Response options were *Yes, I have this*; *I don’t have this, but I wish I had it*; *I don’t have this, but I don’t need it*. Participants were classified as having material deprivation when they responded *I don’t have this, but I wish I had it* to any of the resources.

#### SOGICE

The research team developed the SOGICE items following community consultation about the sorts of practices and language that young people may recognize or use in describing SOGICE practices locally. The question asked, “Have you ever personally experienced ‘conversion therapy’?” with a description: ‘Conversion therapy’ is a practice or treatment that tries to change a person’s sexual orientation or gender or stop them from expressing their rainbow^5^ identity. It is sometimes known as reparative therapy, ex-gay therapy, and healing sexual brokenness. It can also happen in prayer sessions.” Response options were *yes*; *no*; *I’d prefer not to say*. A further question was asked of participants who responded *yes* to the SOGICE question, “Which of the following people suggested ‘conversion therapy’ to you? (Please select all that apply).” Response options included *A leader in my religious or spiritual community*; *A medical professional*; *A family/whānau*^6^*member; myself;* and *another person (please describe)*. Text responses to the *another person* item were reviewed and recoded into the above categories if appropriate. Self-suggested SOGICE was determined by any response that included “myself” as a suggester.

The SOGICE item used in this study focused on who suggested SOGICE to young people, rather than who initiated it. In response to feedback from community consultation when designing the survey, the word “suggest” was used, rather than “told”, to recognize both the overt, as well as covert ways, that people communicated SOGICE to young people. By focusing on who suggested SOGICE the item also recognizes that who suggests SOGICE may be distinct from who conducts or initiates it, and that the advocacy of suggesters may be an important feature in how these practices are subsequently viewed and experienced by the young person.

The wording of this item reflected a range of experiences and nuances in the context of heteronormativity. Some recent research, including a youth-specific study (Green et al., 2020), also combined sexual orientation and gender identity change efforts in the same question. Although separating change efforts by gender or sexual orientation could provide more specificity as to their effects, the operation of the heterosexual matrix (Butler, 1990) means it is not always possible, or even intelligible, to identify whether the change efforts were due to a person’s gender or sexuality. For instance, a cisgender young man’s sexual attraction to other men may result in others’ reframing his gender identity as feminine, in keeping with the heterosexual matrix’s requirement of compulsory ‘opposite’ sex, and therefore gender, attraction. Here he may face policing of his gender (e.g., sissy and ‘fag’ discourses, not being ‘man’ enough, etc.) despite being cisgender, making the distinction for SOGICE based on his sexual orientation versus his gender confounded. While in some situations, a young person may have a clear understanding of a sexual orientation vs gender identity motivation for their SOGICE experience, others may not be able or wish to untangle others’ motivations. The APA recently identified this conflation in their 2021 Resolution on Sexual Orientation Change Efforts, noting that sexual orientation change efforts “…have not only targeted sexual and romantic behavior but also gender expressions that do not conform to stereotypes. In this way, gender identity change efforts have also been a component of SOCE.” (APA, 2021b, p.1).

#### NSSI and suicidality

The following questions were adopted from the Youth2000 survey series, a national youth risk behavior surveillance survey used with high school students in Aotearoa New Zealand (e.g., Fleming et al., 2020). Experiences of NSSI were determined by the question, “In the past 12 months, have you hurt yourself on purpose or done anything you knew might harm you (but not kill you)?” Response options were *No, never*; *Once or twice*; *Three or more times*. To assess suicidality participants were asked, “In the past 12 months, which of the following have you done? (Please select all that apply).” Response options were *Thought about killing yourself* (suicide ideation); *Made a plan about how you would kill yourself* (suicidal plan); *Tried to kill yourself* (suicide attempt).

### Data Analysis

All data analyses were performed in SPSS version 27. Missing values for the Material Deprivation Index (ranging from 0.4 to 15.3%) included participants who selected “I don’t have this, but don’t need it.” Missing values were imputed using the expectation maximization method through the estimation of means and covariances of available data in the index (Enders, 2003).

Differences in proportion for SOGICE exposure across sociodemographic variables were determined using chi-squared goodness-of-fit tests. For dichotomous variables (ethnicity, sexuality, homelessness, and Oranga Tamariki^7^/statutory care experience), the corrected p-value for a 2 × 2 comparison was reported. Next, regression analyses in generalized linear models were performed to identify the associations between SOGICE exposure and different SOGICE suggesters, with NSSI and suicidality. Ordinal regression was employed for NSSI and binary logistic regression for suicide ideation, suicidal plan, and suicide attempt. Covariates in multivariate models were age, gender, and material deprivation. An alpha level of *p* < 0.05 was used to determine statistical significance in all analyses.

## Results

Table 1 displays the proportion of Identify participants who had ever experienced SOGICE. In total, 124 participants (3.1%; 95% confidence interval [CI] 2.6–3.7) reported ever having experienced SOGICE. As presented in Table 2, a higher proportion of those exposed to SOGICE was aged 19–26 years (vs 14–18 years), were trans, non-binary, or another non-cisgender identity, were unsure/questioning gender, and reported severe deprivation. Table 3 outlines the associations between SOGICE exposure and NSSI and suicidality. Participants with SOGICE exposure were more likely than those without SOGICE experience to report NSSI, suicide planning, and suicide attempts. After adjusting for age, gender, and material deprivation, SOGICE exposure was significantly associated with increased NSSI frequency (OR 1.47; 95% CI 1.03–2.08), and more than two times the odds of planning suicide (OR 2.56; 95% CI 1.74–3.78) and attempting suicide (OR 2.73; 95% CI 1.70–4.39).

**Table 2:** Percentage of participants reporting SOGICE across demographic groups (*N* = 3948)

	SOGICE experiences		
Demographic characteristics	Yes (*n*, %)	No (*n*, %)	Chi-square χ^2^ analyses
Total response ethnicity			
Māori	16 (2.8)	553 (97.2)	χ^2^ (1) = 0.11, *p* = 0.737
Samoan	3 (4.1)	70 (95.9)	χ^2^ (1) = 0.02, *p* = 0.883
Cook Island Māori	0	41 (100)	χ^2^ (1) = 0.50, *p* = 0.481
Tongan	2 (11.8)	15 (88.2)	χ^2^ (1) = 1.83, *p* = 0.176
Chinese	3 (1.9)	157 (98.1)	χ^2^ (1) = 0.49, *p* = 0.486
Indian	5 (4.7)	101 (95.3)	χ^2^ (1) = 0.45, *p* = 0.502
Filipino	1 (1.4)	72 (98.6)	χ^2^ (1) = 0.28, *p* = 0.595
Dutch	3 (5.3)	54 (94.7)	χ^2^ (1) = 0.30, *p* = 0.582
British	6 (6.2)	91 (93.8)	χ^2^ (1) = 2.12, *p* = 0.145
Pākehā or New Zealand European	101 (3.0)	3212 (97.0)	χ^2^ (1) = 0.27, *p* = 0.601
Age group^a^			χ^2^ (1) = 13.82, *p* < 0.001
14–18	40 (2.1)	1898 (97.9)	
19–26	84 (4.2)	1926 (95.8)	
Gender groups			χ^2^ (3) = 15.83, *p* = 0.001
Cisgender	38 (2.0)	1835 (98.0)	
Trans man and trans woman	25 (4.5)	534 (95.5)	
Nonbinary and another gender	43 (4.4)	943 (95.6)	
Unsure	18 (3.5)	503 (96.5)	
Total response Sexuality			
Heterosexual/straight	2 (5.6)	34 (94.4)	χ^2^ (1) = 0.13, *p* = 0.723
Mostly straight	1 (0.9)	112 (99.1)	χ^2^ (1) = 1.26, *p* = 0.262
Takatāpui	12 (5.1)	222 (94.9)	χ^2^ (1) = 2.57, *p* = 0.109
Queer	66 (3.6)	1749 (96.4)	χ^2^ (1) = 2.40, *p* = 0.121
Gay	31 (3.3)	895 (96.7)	χ^2^ (1) = 0.09, *p* = 0.763
Lesbian	27 (3.5)	745 (96.5)	χ^2^ (1) = 0.27, *p* = 0.606
Bisexual	48 (2.8)	1663 (97.2)	χ^2^ (1) = 0.94, *p* = 0.332
Pansexual	30 (3.2)	894 (96.8)	χ^2^ (1) = 0.01, *p* = 0.920
Asexual	24 (4.2)	547 (95.8)	χ^2^ (1) = 2.08, *p* = 0.150
Aromantic	4 (2.4)	164 (97.6)	χ^2^ (1) = 0.12, *p* = 0.725
Demisexual	12 (3.2)	364 (96.8)	χ^2^ (1) = 0.00, *p* = 1.00
Fluid/it changes	17 (2.9)	566 (97.1)	χ^2^ (1) = 0.04, *p* = 0.833
Unsure	8 (2.6)	301 (97.4)	χ^2^ (1) = 0.17, *p* = 0.681
Material Deprivation			χ^2^ (2) = 27.06, *p* < 0.001
No deprivation (0)	59 (2.7)	2158 (97.3)	
Mild deprivation (1–4)	53 (3.3)	1566 (96.7)	
Severe deprivation (5–9)	12 (11.9)	89 (88.1)	
Homelessness	41 (11.0)	331(89.0)	χ^2^ (1) = 82.29, *p* < 0.001
Staturory Care/Oranga Tamariki involvement^a^	20 (5.1)	369 (94.9)	χ^2^ (1) = 5.05, *p* = 0.025

^a^The *p*-value for continuity correction for a 2 × 2 comparison is reported to avoid over-estimation of chi-squared values

**Table 3:** Association between SOGICE and NSSI and suicidality (*n* = 3948)

	NSSI^a^		Suicide Ideation^b^		Suicide Planning^b^		Suicide Attempt^b^	
	Bivariate	Multivariate	Bivariate	Multivariate	Bivariate	Multivariate	Bivariate	Multivariate
SOGICE	1.58 (1.14–2.21)**	1.47 (1.03–2.08)*	1.48 (0.99–2.20)	1.24 (0.81–1.89)	2.60 (1.82–3.73)***	2.56 (1.74–3.78)***	3.12 (2.03–4.80)***	2.73 (1.70–4.39)***

Multivariate models adjusted for age, gender, and material deprivation. Data are presented as odds ratio (95% confidence interval)

*NSSI* non-suicidal self-injury, *SOGICE* sexual orientation and gender identity change efforts

^a^Ordinal logistic regression

^b^Binary logistic regression

*p < 0.05, **p < 0.01, ***p < 0.001

Participants reporting SOGICE exposure had SOGICE suggested by family/whānau members (55.6%), religious or spiritual leaders (48.4%), myself (17.7%), another person (14.5%), and medical professionals (11.3%). Of the 22 participants who selected “myself,” some also selected religious leaders (*n* = 11; 50.0%), family members (*n* = 11; 50.0%), medical professionals (*n* = 3; 13.6%), or another person (*n* = 3; 13.6%), and nine selected “myself” only (41.0%). Table 4 presents the association between SOGICE exposure from each suggester type and NSSI and suicidality. In bivariate models, those who experienced SOGICE suggestion from religious or spiritual leaders had a higher likelihood of reporting a suicide plan and suicide attempt. Similar findings were observed for medical professionals and family/whānau members as SOGICE suggesters. However, significantly higher NSSI frequency and suicide ideation were found amongst participants whose family/whānau members suggested SOGICE to them. In multivariate models that adjusted for age, gender, and material deprivation, those exposed to SOGICE-suggestion from religious or spiritual leaders were significantly more likely to engage in NSSI, suicidal planning, and suicide attempts (OR 3.00; 95% CI 1.54–5.84). Having family/whānau members who suggested SOGICE was also a significant predictor for suicidal planning and at least one suicide attempt (OR 2.27; 95% CI 1.20–4.31).

**Table 4:** Association between SOGICE suggester and NSSI and suicidality (*n* = 3948)

	NSSI^a^		Suicide Ideation^b^		Suicide Planning^b^		Suicide Attempt^b^	
	Bivariate	Multivariate	Bivariate	Multivariate	Bivariate	Multivariate	Bivariate	Multivariate
Religious leaders	1.62 (1.00–2.63)	1.71 (1.03–2.83)*	1.36 (0.78–2.38)	1.22 (0.68–2.19)	2.43 (1.46–4.05)***	2.70 (1.56–4.67)***	2.98 (1.62–5.48)***	3.00 (1.54–5.84)**
Medical professionals	2.56 (0.96–6.82)	1.72 (0.62–4.82)	3.50 (0.78–15.66)	2.11 (0.44–10.10)	3.42 (1.19–9.89)*	2.47 (0.78–7.82)	2.62 (0.73–9.43)	1.21 (0.29–5.02)
Family/whānau member	1.63 (1.05–2.53)*	1.44 (0.91–2.29)	1.76 (1.01–3.06)*	1.42 (0.79–2.54)	2.78 (1.71–4.47)***	2.57 (1.53–4.32)***	3.03 (1.71–5.36)***	2.27 (1.20–4.31)*
Myself (self-suggested SOGICE)	1.53 (0.70–3.36)	1.70 (0.76–3.82)	1.98 (0.73–5.39)	1.81 (0.64–5.12)	2.57 (1.11–5.94)*	2.92 (1.20–7.08)*	3.63 (1.41–9.32)**	3.86 (1.39–10.66)**
Another person	1.56 (0.66–3.65)	1.16 (0.48–2.80)	1.16 (0.44–3.11)	0.82 (0.29–2.28)	2.05 (0.81–5.21)	1.57 (0.59–4.19)	2.75 (0.90–8.40)	2.20 (0.68–7.16)
Number of categories of suggesters [0–2] - 0, 1, 2, or more^c^	1.37 (1.08–1.74)**	1.29 (1.01–1.66)*	1.33 (1.00–1.78)	1.17 (0.86–1.58)	1.81 (1.40–2.34)***	1.77 (1.34–2.33)***	1.92 (1.42–2.60)***	1.71 (1.22–2.40)**

Multivariate models adjusted for age, gender, and material deprivation. Data are presented as odds ratio (95% confidence interval)

*NSSI* non-suicidal self-injury, *SOGICE* sexual orientation and gender identity change efforts

^a^Ordinal logistic regression

^b^Binary logistic regression

^c^Including medical professional, family/whānau member, religious leader, another person

*p < 0.05, **p < 0.01, ***p < 0.001

Participants who reported self-suggesting SOGICE were separated from those who had been exposed to SOGICE-suggestion by other individuals. In the sample of those exposed to other-suggested SOGICE (*n* = 114), 61.4% (*n* = 70) of participants had reported one type of SOGICE-suggester, and 38.6% (*n* = 44) were exposed to two or more types of SOGICE suggesters. In bivariate models, participants exposed to increasing types of SOGICE suggesters had a higher likelihood of engaging in NSSI and all the suicidality variables that were examined. After adjusting for sociodemographic variables, an increment of exposure to one SOGICE-suggester (on a scale of 0 to 2), on average, was significantly associated with 29% increased odds of NSSI frequency, 77% for a suicidal plan, and 71% for a suicide attempt.

## Discussion

Studies on SOGICE, predominantly with adult populations, have demonstrated associations with adverse mental health outcomes and highlight that particular groups may be at higher risk of harm. However, few of these studies have been explicitly designed to explore young people’s experiences. Young people may face additional challenges when exposed to SOGICE, as adolescence is the time when identity development and consolidation are central to positive youth development. The effects of SOGICE that seek to change a key part of young people’s identity at this developmental stage may therefore be particularly harmful. Relationships are fundamental to identity development; however, there is limited research on whether particular categories of people who suggest SOGICE to young people are differentially associated with harm. This study addressed these gaps by collecting mental health and demographic data, including data on who suggested SOGICE to participants. The results confirmed that SOGICE is occurring for an important minority of young people in New Zealand and that it is often very harmful. Mental health impacts did vary according to who suggested SOGICE to the young person, the frequency of categories of people who suggested it, and young people’s membership in particular demographic groups that were associated with increased stigma and distress.

The findings indicate that at least 3.0% of a contemporary youth sample have experienced SOGICE. Studies using alternative measures targeting particular SOGICE behaviors or phenomena have produced higher estimates among Canadian participants (Salway et al., 2021) and transgender and gender-diverse participants in New Zealand (Veale et al., 2021). One potential explanation for the relatively low reporting of SOGICE in the present study could be due to the wording of the question around SOGICE. The question in the current study was written to describe experiences and use language that was likely to be recognizable to the local younger age group of the study. However, the question wording may have emphasized direct SOGICE experiences rather than indirect processes or the withholding of services to enforce a change in identity. As a measure, this may have produced a more conservative estimate of prevalence than measures that explicitly include SOGICE through indirect means (e.g., the denial of appropriate health care or services based on gender identity, etc.) (Veale et al., 2021). In addition, the question in the current study initially required participants to endorse that their experience was “conversion therapy.” Although the wording of the question described a range of “practices,” the initial use of the word therapy may have made it difficult for participants to reconcile a negative experience with something that was purported to be “therapeutic.”. As such, when including participants who reported they were too upset to answer the initial question or who said they preferred not to respond once asked the question (see Appendix Table 5), the prevalence rose to 7.1%, which may be a more accurate estimate of prevalence in a nonprobability youth sample.

The study shows that older youth were more likely to report SOGICE. In this way, the study reflects other research predominantly with adults showing increased age-related prevalence in Hong Kong (Chan et al., 2022) and South Korea (Lee et al., 2021) but differs from recent US (Green et al., 2020) and NZ (Veale et al., 2021) research showing greater prevalence in younger participants. The age differences in the current study may indicate that older youth may have had more time to understand and recognize their SOGICE experiences, and may therefore be more likely to report such experiences. In addition, older youth may be more willing to answer questions about experiences that may now be historic and potentially less distressing than younger participants who may currently have, or only recently had, SOGICE experiences. The findings show that younger youth were more likely to skip or decline to answer these questions (see Appendix Table 5), which might mean that SOGICE experiences for younger youth are underrepresented in the study. Internationally, more research with youth populations is required to understand these trends and acknowledge that many presently enduring such experiences may be too distressed to answer these items, leading to underreporting among younger youth.

Critically, the results align with findings from international literature that show an association between SOGICE and NSSI and suicidality among adults, as well as among young people aged ≤24 years (Green et al., 2020). The odds for suicide attempts in the past year were nearly three times for participants who had experienced SOGICE compared with those who had not; this effect size was similar to the findings observed among gender- and sexuality-diverse youth exposed to SOGICE in the USA (Green et al., 2020). The results are consistent with the hypothesis that the effects of SOGICE are particularly damaging to young people’s mental health. The higher risk of NSSI, suicide planning, and suicide attempts in the past year highlights that experiences of SOGICE that may have occurred earlier in life, even in the relatively short lives of young people, may produce long-term harm. SOGICE practices are associated with life-threatening implications, emphasizing the importance of prohibiting such practices and providing trauma-informed mental health support, including for historical experiences.

The findings show that the prevalence of SOGICE varies by some sociodemographic features and not others. For instance, SOGICE was relatively equally reported by participants across all ethnicities and sexualities but was more likely to be reported by young people who were transgender, non-binary or another gender, or unsure whether they were transgender, than by cisgender young people. The higher rates reported by transgender and gender-diverse young people could indicate that they may have experienced change efforts directed at their gender identity *and* their sexual orientation, and this may increase the potential for distress. Service provision and future research should recognize those young people who are both gender- and sexuality-diverse may face increased challenges compared to those who occupy only one of these identities.

Additionally, transgender and gender-diverse young people may affirm their non-cisgender identities at an early age (Fast & Olson, 2018) than sexuality-diverse young people do their non-heterosexual identities (Bishop et al., 2022). In this way, they may face increased exposure to earlier SOGICE than sexuality-diverse young people, and this earlier age of SOGICE exposure, or the increased duration of SOGICE exposure, may also explain the increased associations of SOGICE-related harm for these young people. Future research is required to explore whether the age of recognizing one’s gender- and sexuality-diverse identity, alongside the age of first SOGICE exposure, are related, and potentially associated with mental health outcomes.

Transgender and gender-diverse young people may also be more likely to face SOGICE in healthcare settings than sexuality-diverse young people because accessing gender-affirming care requires the disclosure of a gender-diverse identity. Disclosing a gender-diverse identity may increase the chances that these young people face SOGICE by healthcare practitioners. The study shows that family members are often involved in suggesting SOGICE to transgender and gender-diverse young people. One way that they may do this is to delay, discourage and prevent access to gender-affirming health care. The study underscores that transgender and gender-diverse young people need stronger protections and must have access to unbiased gender-affirming healthcare without cisheteronormative gatekeeping by health care practitioners or parents and guardians. The study emphasizes that transgender and gender-diverse young people must be explicitly named and visible in SOGICE policy, intervention, and practice outcomes to ensure that their unique and increased needs are addressed.

Young people reporting severe deprivation were also more likely to report SOGICE than those reporting less deprivation. While this may be a byproduct of the increased rates of homelessness and statutory care experience reported by those who reported SOGICE experiences, it nonetheless emphasizes the need for accessible and affordable mental health supports for gender- and sexuality-diverse young people and adults. Accessible and affordable mental health care support is critical in this situation, as those reporting severe deprivation may not be able to afford treatment otherwise.

Equally, policies and processes for young people who are homeless or have experienced homelessness or involvement with statutory child services must recognize that SOGICE experiences are statistically more common for this group and respond accordingly. A range of research demonstrates the overrepresentation of gender- and sexuality-diverse young people experiencing homelessness or statutory care (Baams, 2019). SOGICE may be a direct or indirect cause of homelessness or statutory care experience, which means that secular statutory care services may be essential for young people who may have been traumatized by religious SOGICE experiences. The study could not determine whether family members who suggested SOGICE were foster parents or siblings or whether the young people were in statutory care because of such experiences from family members. Given the prevalence of young people in care reporting SOGICE experiences, statutory care workers must be educated on the damaging effects of SOGICE and screened to ensure such practices are avoided in future.

A sensitivity analysis explored whether a similar pattern of sociodemographic characteristics was associated with reluctance to answer the SOGICE items. Appendix Table 5 shows that other than the noted higher prevalence among younger young people to skip the SOGICE section, the other differences were higher rates of skipping by nonbinary young people, Māori, and Cook Island Māori participants. Sample sizes were small in this analysis, so caution is required when drawing comparisons; however, future research is needed to understand why participants from these groups were more likely to skip these questions. It may be that more members of these groups experienced current or recent SOGICE exposure compared to participants from other groups in the study. However, the higher rates of non-binary youth who declined to answer SOGICE questions, alongside the higher rates of non-binary young people who reported SOGICE when they did answer these items, highlights a concerning trend for this group. One possible reason for the overrepresentation of non-binary participants in both sets of these results may reflect the largely transnormative nature of gender-affirming care (Pasley et al., 2022) that may place non-binary populations at greater risk of SOGICE compared to young people with other gender identities. More research on these populations, as well as continued validation and improved understandings of Māori, Cook Island Māori and non-binary identities, are important opportunities that may improve outcomes.

A wide range of people may suggest SOGICE to a young person, including family members. The study found a strong association between family member-suggested SOGICE and suicidality, which reflects USA research showing that participants whose parents had initiated sexual orientation change efforts had three times the odds of reporting a suicide attempt than those who had not experienced this (Ryan et al., 2020). The significance of family members’ suggestions of SOGICE may reflect the fact that the majority of young people rely on family members for their survival and wellbeing, including meeting their housing, living and financial needs. Many family members will also meet many of the emotional needs of young people. Therefore, when such family members, who often have a lot of power and influence on young people, suggest SOGICE, it may be more distressing than when other, less powerful and intimately aquatinted adults and peers suggest SOGICE. In contrast, other research has highlighted that families’ positive responses to their child’s gender or sexual orientation can foster positive outcomes in health (Newcomb et al., 2019), education (Fenaughty et al., 2019), and self-identity (Katz-Wise et al., 2016). These effects emphasize the need for family education and support, particularly around the negative impacts of cisheteronormative rejection (American Psychological Association, 2021a) and the reconciliation of religion and LGBTQIA+ identities (VanderWaal et al., 2017).

SOGICE suggested by religious leaders was also significantly more likely to be associated with higher NSSI and suicide planning and attempts in the past year. The significance of religious leaders’ role in SOGICE-related harm may reflect that such leaders are often highly trusted by young people and their families. When such people, who ostensibly have young people’s best interests at heart, suggest SOGICE, a disproven therapeutic activity (American Psychological Association, 2021b), this results in significantly increased odds of suicidality and NSSI. Prohibiting people in such roles from suggesting or initiating SOGICE is an essential public health response to protect gender- and sexuality-diverse young people from harm. Alongside education for parents and families, policy and practice interventions are required to support the education of religious and spiritual communities about the harm associated with SOGICE to help prevent such experiences from continuing.

People who self-suggest SOGICE may be particularly negative about their diverse gender and sexuality. Some of the increased harm reported for self-suggestion of SOGICE may represent the increased anguish and shame at “failing” not only themselves but also others when the promises of SOGICE are not realized. Understanding young people’s self-suggestion within the context of the cisheteronormative beliefs and norms that influence youth development is critical. Although only a minority of gender- and sexuality-diverse young people self-suggest SOGICE, such decisions are hazardous and associated with the most harm of any group in the study. Prohibiting SOGICE experiences, even when self-suggested, is a critical public health recommendation from this study, and young people who seek out such experiences are an urgent priority for mental health and support services. The increased risk of NSSI and suicidality for young people who self-suggest SOGICE also strengthens and broadens the recommendations for education and support on this issue. A public health response requires that all young people, irrespective of their gender or sexuality, receive comprehensive education about gender and sexuality diversity to counter destructive norms that devalue and stigmatize their identities (Fenaughty, 2019).

Half of the young people who self-suggested SOGICE also reported that another person had suggested SOGICE to them, indicating that others’ attitudes and beliefs often inform self-suggestion decisions. Further, Table 4 shows that harm increased as the frequencies of categories of suggesters increased. The increased risk associated with multiple types of SOGICE-suggesters underscores the power of others to influence or mitigate SOGICE self-suggestion and initiation. Further studies are required to determine the nuances within the relationship between the number of SOGICE-suggester types and NSSI and suicidality risks; nonetheless, mental health services are advised to screen for the frequency of types of suggesters of SOGICE and to consider the increased risk of harm for young people who report more than one type of suggester.

Sample bias is one of the crucial limitations of a nonprobability sample. Recruitment for the study relied on the internet and social media, as well as regional libraries, mass media stories, and posters in schools and tertiary education providers. The call to participate in the research was also widely shared through rainbow community networks and media. Young people connected to rainbow communities and media may therefore have been more likely to see the call to participate. Such young people may differ from those not connected to rainbow communities and media, as they may have more rainbow-friendly social connections and supports, which may operate as protective factors for their mental health. The greater concentration of more-connected participants in the study means the data may underestimate the adverse effects of SOGICE experiences because it cannot account for those who have fewer connections and, therefore, less supports. Young people currently undergoing SOGICE may also have been less likely to be engaging with rainbow communities and media, and they may have been less likely to see the call to participate, resulting in a potential underestimate of the prevalence of SOGICE. Nonetheless, a key strength is the large sample size, which enabled comparisons *within* the sample to tease out effects while accounting for bias to the greatest degree possible.

The research literature and professional guidelines emphasize that SOGICE is ineffective and harmful (American Psychological Association, 2021a). It is doubtful that a large population of “converted” young people who are satisfied with their SOGICE experience exist or report positive mental health outcomes due to SOGICE. The literature demonstrates that SOGICE is associated with adverse short- and long-term mental health outcomes, including suicidality. Extrapolating these mental health findings means that many young people with SOGICE exposure may experience debilitating mental health outcomes and may not be able, or even alive, to participate in research on SOGICE exposure. As such, all cross-sectional SOGICE survey findings are more likely to underestimate the prevalence and adverse effects of SOGICE.

The question wording may have inadvertently created barriers to young people reporting a negative “therapy” experience. A question focusing on practices and withholding services may generate a higher prevalence of SOGICE, including by health practitioners, who were less implicated than in other studies. While the adverse effects of SOGICE in the study were comparable to international studies, the prevalence was lower. For these reasons, the prevalence data on young people who said they were too upset to answer the SOGICE item or said they preferred not to answer further questions on SOGICE after the initial question is included (see Appendix Table 5). The combined proportion of participants who requested to skip this question due to being upset, alongside those who did report SOGICE, may better represent those who experienced SOGICE.

As a cross-sectional study, the findings are correlational and do not prove causality; however, these findings are consistent with the literature detailing the harmful effects of SOGICE among mainly adult participants, as well as a large non-probability youth sample in the US (Green et al., 2020), and qualitative findings (Kinitz et al., 2021). Furthermore, the outcome measures of NSSI, suicide plans and suicide attempts were limited to the past 12 months. It is implausible that all of the SOGICE practices in this study happened in the past year alone, and these data suggest the potential for long-term harm that occurs after SOGICE exposure in young people. Further research is required to understand how SOGICE is associated with mental health problems over time, which might explore whether there are compounding effects of SOGICE over time.

The analysis presented focuses on gender- and sexuality-diversity but not on variations in sex characteristics. While intersex young people were included in the analysis, the sample size was too small to separately analyze young people with variations in sex characteristics. Further research is required to explore key factors for young people with variations in sex characteristics in relation to SOGICE.

## Conclusion

Adolescence is characterized as a period of significant identity development, yet few youth-specific studies have explored how SOGICE experiences affect people at this age. Using a survey methodology with a large sample of gender- and sexuality-diverse young people, this study shows that SOGICE occurs in youth and demonstrates important demographic and mental health associations. Building on recent research on adult populations, this study emphasizes the additional challenges facing transgender and gender-diverse young people, who experience higher rates of SOGICE suggestion. The findings highlight the increased risk of SOGICE for young people experiencing material deprivation, homelessness, and statutory care. The study extends the field by identifying that SOGICE suggested by religious leaders and family/whānau members were associated with the highest occurrence of reported NSSI, suicidal planning and suicide attempts. SOGICE suggestions by people whom youth may respect, value, and rely on, like religious leaders and family members, may be particularly harmful to their positive identity development and subsequent mental health. Increased rates of harm were also associated with reports of multiple categories of people suggesting SOGICE or self-suggested SOGICE. In addition to prohibiting SOGICE, particularly when suggested by religious leaders and family members, free and accessible mental health support is a priority for those exposed to SOGICE. Particular groups require additional support, including transgender, non-binary and gender-diverse young people, statutory care-experienced youth, and young people who have experienced homelessness. Education for families, religious leaders and communities, health and social service providers, and all young people, regardless of their gender and sexuality identities, is a critical public health response to address the stigmatizing attitudes, beliefs, and norms that enable SOGICE suggestion and initiation to continue.

## Appendix

Table 5

**Table 5:** Percentage of participants who said they were too upset, or actively declined, to answer SOGICE questions, across demographic groups (*N* = 4118)

	SOGICE experiences		
Demographic characteristics	Found the topic upsetting or said prefer not to say (*n*, %)	Yes (*n*, %)	Chi-square χ^2^ analyses
Total response ethnicity^a^			
Māori (*n* = 608)	39 (6.4)	16 (2.6)	χ^2^ (2) = 9.49, *p* = 0.009
Samoan (*n* = 79)	6 (7.6)	3 (3.8)	χ^2^ (2) = 2.65, *p* = 0.266
Cook Island Māori (*n* = 46)	5 (10.9)	0 (0.0)	χ^2^ (2) = 6.55, *p* = 0.038
Tongan (*n* = 19)	2 (10.5)	2 (10.5)	χ^2^ (2) = 5.88, *p* = 0.053
Chinese (*n* = 170)	10 (5.9)	3 (1.8)	χ^2^ (2) = 2.20, *p* = 0.333
Indian (*n* = 108)	2 (1.9)	5 (4.6)	χ^2^ (2) = 2.40, *p* = 0.302
Filipino (*n* = 75)	2 (2.7)	1 (1.3)	χ^2^ (2) = 1.19, *p* = 0.552
Dutch (*n* = 58)	1 (1.7)	3 (5.2)	χ^2^ (2) = 1.76, *p* = 0.415
British (*n* = 102)	5 (4.9)	6 (5.9)	χ^2^ (2) = 3.20, *p* = 0.202
Pākehā or New Zealand European (*n* = 3441)	128 (3.7)	101 (2.9)	χ^2^ (2) = 9.99, *p* = 0.007
Age group^a^			χ^2^ (2) = 29.23, *p* < 0.001
14–18 (*n* = 2047)	109 (5.3)	40 (2.0)	
19–26 (*n* = 2071)	61 (2.9)	84 (4.1)	
Gender groups			χ^2^ (6) = 24.02, *p* < 0.001
Cisgender (*n* = 1941)	68 (3.5)	38 (2.0)	
Trans man and trans woman (*n* = 578)	19 (3.3)	25 (4.3)	
Non-binary and another gender (*n* = 1043)	57 (5.5)	43 (4.1)	
Unsure (*n* = 547)	26 (4.8)	18 (3.3)	
Total response Sexuality			
Heterosexual/Straight (*n* = 36)	0 (0)	2 (5.6)	χ^2^ (2) = 2.29, *p* = 0.318
Mostly straight (*n* = 115)	2 (1.7)	1 (0.9)	χ^2^ (2) = 3.70, *p* = 0.157
Takatāpui (*n* = 249)	15 (6.0)	12 (4.8)	χ^2^ (2) = 5.56, *p* = 0.062
Queer (*n* = 1884)	69 (3.7)	66 (3.5)	χ^2^ (2) = 4.62, *p* = 0.099
Gay (*n* = 965)	39 (4.0)	31 (3.2)	χ^2^ (2) = 0.19, *p* = 0.908
Lesbian (*n* = 804)	32 (4.0)	27 (3.4)	χ^2^ (2) = 0.46, *p* = 0.797
Bisexual (*n* = 1771)	60 (3.4)	48 (2.7)	χ^2^ (2) = 5.46, *p* = 0.065
Pansexual (*n* = 964)	40 (4.1)	30 (3.1)	χ^2^ (2) = 0.04, *p* = 0.978
Asexual (*n* = 597)	26 (4.4)	24 (4.0)	χ^2^ (2) = 2.55, *p* = 0.279
Aromantic (*n* = 176)	8 (4.5)	4 (2.3)	χ^2^ (2) = 0.41, *p* = 0.813
Demisexual (*n* = 392)	16 (4.1)	12 (3.1)	χ^2^ (2) = 0.01, *p* = 0.997
Fluid/It changes (*n* = 608)	25 (4.1)	17 (2.8)	χ^2^ (2) = 0.12, *p* = 0.944
Unsure (*n* = 321)	12 (3.7)	8 (2.5)	χ^2^ (2) = 0.47, *p* = 0.789
Material Deprivation			χ^2^ (4) = 64.26, *p* < 0.001
No deprivation (0) (*n* = 2279)	62 (2.7)	59 (2.6)	
Mild deprivation (1–4) (*n* = 1707)	88 (5.2)	53 (3.1)	
Severe deprivation (5–9) (*n* = 116)	15 (12.9)	12 (10.3)	
Homelessness (*n* = 394)	22 (5.6)	41 (10.4)	χ^2^ (2) = 86.94, *p* < 0.001
Staturory Care/Oranga Tamariki involvement^a^ (*n* = 419)	30 (7.2)	20 (4.8)	χ^2^ (2) = 19.44, *p* < 0.001

^a^The *p*-value for continuity correction for a 2 × 2 comparison is reported to avoid over-estimation of chi-squared values

## Footnotes

1 This term refers to young people who are positioned, or identify, in diverse ways outside of dominant cisgender and heterosexual norms. The use of this term here includes Indigenous and culturally specific knowledge of gender and sexuality, while acknowledging this term is limited in recognising intersecting aspects and histories of identity (Mizzi & Walton, 2014). The term is used here to emphasise cisheterosexism and its effects, yet other oppressions are obscured in this framing.

2 Takatāpui is a traditional Māori term (the indigenous language of Aotearoa New Zealand), originally meaning “intimate partner of the same sex” (Kerekere, 2015). It has been reclaimed and is now used to describe Māori who are gender or sexuality-diverse or who are born with variations in sex characteristics.

3 MVPFAFF+ is an acronym that encompasses Pacific expressions of gender and sexuality. It stands for Mahu (Hawai’i and Tahiti), Vaka sa lewa lewa (Fiji), Palopa (Papua New Guinea), Fa’afafine (Samoa), Akava’ine (Rarotonga), Fakaleitī (Tonga), and Fakafifine (Niue) (New Zealand Human Rights Commission, 2020).

4 A Māori word that describes White settlers and immigrants of European descent.

5 The term “rainbow” was used throughout the Identify Survey as an umbrella term to include gender- and sexuality-diverse and intersex people. It is a term increasingly used in Aotearoa New Zealand to refer to queer, trans, and intersex communities.

6 Whānau is a Māori word that loosely translates to family; however, family in this context often includes extended family (i.e., beyond parents and siblings, to include grandparents, parents’ siblings, and cousins), and sometimes close friends.

7 Oranga Tamariki is the government agency responsible for ensuring the wellbeing of tamariki (children), rangatahi (youth), and whānau (families) in Aotearoa New Zealand. It is the statutory body that can remove children and young people from their families and place them into residential or foster care.

## References

[CR1] American Psychological Association. (2021a). *APA resolution on gender identity change efforts*. https://www.apa.org/about/policy/resolution-gender-identity-change-efforts.pdf.

[CR2] American Psychological Association. (2021b). *APA resolution on sexual orientation change efforts*. https://www.apa.org/about/policy/resolution-sexual-orientation-change-efforts.pdf.

[CR3] Baams L, Wilson BD, Russell ST (2019). LGBTQ youth in unstable housing and foster care. Pediatrics.

[CR4] BBC. (2020, May 8). *Germany passes law banning ‘gay conversion therapy’ for minors*. BBC. https://www.bbc.com/news/world-europe-52585162.

[CR5] Bishop, M. D., Mallory, A. B. & Russell, S. T. (2022). Sexual minority identity development. *Psychology of Sexual Orientation and Gender Diversity, Publish Ahead of Print*, 10.1037/sgd0000569. 10.1037/sgd0000569 PMC10756425 38162689

[CR6] Blosnich JR, Henderson ER, Coulter RW, Goldbach JT, Meyer IH (2020). Sexual orientation change efforts, adverse childhood experiences, and suicide ideation and attempt among sexual minority adults, United States, 2016–2018. American Journal of Public Health.

[CR7] Butler, J. (1990). *Gender trouble: Feminism and the subversion of identity*. Routledge.

[CR8] Chan, R. C. H., Leung, J. S. Y., & Wong, D. C. K. (2022). Experiences, motivations, and impacts of sexual orientation change efforts: Effects on sexual identity distress and mental health among sexual minorities. *Sexuality Research and Social Policy*. 10.1007/s13178-021-00669-5.

[CR9] Cowie L, Braun V (2021). Between social and biomedical explanation: queer and gender-diverse young people’s explanations of psychological distress. Psychology and Sexuality.

[CR10] Enders CK (2003). Using the expectation maximization algorithm to estimate coefficient alpha for scales with item-level missing data. Psychological Methods.

[CR11] Fast AA, Olson KR (2018). Gender development in transgender preschool children. Child Development.

[CR12] Fenaughty J (2019). Developing resources to address homophobic and transphobic bullying: a framework incorporating co-design, critical pedagogies, and bullying research. Sex Education.

[CR13] Fenaughty J, Lucassen MF, Clark T, Denny S (2019). Factors associated with academic achievement for sexual and gender minority and heterosexual cisgender students: implications from a nationally representative study. Journal of Youth and Adolescence.

[CR14] Fenaughty, J., Clark, T., Choo, W.L., Lucassen, M., Greaves, L., Sutcliffe, K., Ball, J., Ker, A., & Fleming, T. (2021b). *Te āniwaniwa takatāpui whānui: Te aronga taera mō ngā rangatahi | Sexual attraction and young people’s wellbeing in Youth19*. Youth19 Research Group. https://www.youth19.ac.nz/s/Same-and-Multiple-Sex-Attracted_030622.pdf.

[CR15] Fenaughty, J., Sutcliffe, K., Fleming, T., Ker, A., Lucassen, M., Greaves, L., & Clark, T. (2021a). Transgender and diverse gender students: A Youth19 brief. Youth19 Survey. https://www.youth19.ac.nz/publications/transgender-and-diverse-students-brief.

[CR16] Fleming, T., Peiris-John, R., Crengle, S., Archer, D., Sutcliffe, K., Lewycka, S., & Clark, T. (2020). *Youth19 Rangatahi Smart Survey Initial Findings: Introduction and Methods*. Youth19 Research Group. https://www.tydt.org.nz/s/Youth19InitialFindingsIntroandMethod.pdf.

[CR17] Green AE, Price-Feeney M, Dorison SH, Pick CJ (2020). Self-reported conversion efforts and suicidality among US LGBTQ youths and young adults, 2018. American Journal of Public Health.

[CR18] Higbee M, Wright ER, Roemerman RM (2020). Conversion therapy in the Southern United States: Prevalence and experiences of the survivors. Journal of Homosexuality.

[CR19] Horne, S. G., & McGinley, M. (2022). Sexual orientation change efforts and gender identity change efforts in international contexts: Global exports, local commodities. In D. C. Haldeman (Ed.), *The case against conversion “therapy”: Evidence, ethics, and alternatives* (pp. 221–246). American Psychological Association. http://www.jstor.org/stable/j.ctv27tcv0h.15.

[CR20] Katz-Wise SL, Rosario M, Tsappis M (2016). Lesbian, gay, bisexual, and transgender youth and family acceptance. Pediatric Clinics.

[CR21] Kerekere, E. (2015). *Takatāpui: Part of the Whānau*. Tīwhanawhana Trust and Mental Health Foundation. https://mentalhealth.org.nz/resources/resource/takatapui-part-of-the-whanau.

[CR22] Kinitz, D. J., Goodyear, T., Dromer, E., Gesink, D., Ferlatte, O., Knight, R., & Salway, T. (2021). “Conversion therapy” experiences in their social contexts: a qualitative study of sexual orientation and gender identity and expression change efforts in Canada. *The Canadian Journal of Psychiatry*. 10.1177/07067437211030498. 10.1177/07067437211030498 PMC9152241 34242106

[CR23] De Lange J, Baams L, Van Bergen DD, Bos HMW, Bosker RJ (2022). Minority stress and suicidal ideation and suicide attempts among LGBT adolescents and young adults: a meta-analysis. LGBT Health.

[CR24] Lee H, Streed CG, Yi H, Choo S, Kim S-S (2021). Sexual orientation change efforts, depressive symptoms, and suicidality among lesbian, gay, and bisexual adults: a cross-sectional study in South Korea. LGBT Health.

[CR25] Lee H, Operario D, Restar AJ, Choo S, Kim R, Eom Y-J, Yi H, Kim S-S (2022). Gender identity change efforts are associated with depression, panic disorder, and suicide attempts in South Korean transgender adults. Transgender Health.

[CR26] Méndez, J. E. 2013. *Report of the special rapporteur on torture and other cruel, or degrading treatment or punishment*. Human Rights Council. https://www.ohchr.org/documents/hrbodies/hrcouncil/regularsession/session22/a.hrc.22.53_english.pdf

[CR27] Mendos, L. R. (2020). Curbing deception: a world survey on legal regulation of so-called ‘conversion therapies’. Geneva: ILGA World. https://ilga.org/Conversion-therapy-global-research-ILGA-World.

[CR28] Ministry of Health (2017). *New Zealand health research strategy 2017–2027*. https://www.health.govt.nz/system/files/documents/publications/nz-health-research-strategy-jun17.pdf.

[CR29] Mizzi R, Walton G (2014). Catchalls and conundrums: Theorizing “Sexual Minority” in social, cultural, and political contexts. Paideusis: Journal of the Canadian Philosophy of Education Society.

[CR30] New Zealand Human Rights Commission (2020). *PRISM: Human rights issues relating to sexual orientation, gender identity and expression, and sex characteristics (SOGIESC) in Aotearoa New Zealand - A report with recommendations*. https://www.hrc.co.nz/files/9215/9253/7296/HRC_PRISM_SOGIESC_Report_June_20.

[CR31] Newcomb, M. E., Lasala, M. C., Bouris, A., Mustanski, B., Prado, G., Schrager, S. M., & Huebner, D. M. (2019). The Influence of Families on LGBTQ Youth Health: A Call to Action for Innovation in Research and Intervention Development. 6(4). 10.1089/lgbt.2018.0157. 10.1089/lgbt.2018.0157 PMC6551980 30844341

[CR32] Pascoe CJ (2005). “Dude, you’re a fag”: adolescent masculinity and the fag discourse. Sexualities.

[CR33] Pasley, A., Hamilton, T., & Veale, J. F. (2022). Transnormativities: Reterritorialising perceptions and practice. In L. Johnston & P. Doan (Eds.), *Rethinking transgender identities (Vol. 1)*. Routledge.

[CR34] Plöderl M, Tremblay P (2015). Mental health of sexual minorities. A systematic review. International Review of Psychiatry.

[CR35] Przeworski A, Peterson E, Piedra A (2021). A systematic review of the efficacy, harmful effects, and ethical issues related to sexual orientation change efforts. Clinical Psychology: Science and Practice.

[CR36] Ryan C, Toomey RB, Diaz RM, Russell ST (2020). Parent-initiated sexual orientation change efforts with LGBT adolescents: Implications for young adult mental health and adjustment. Journal of Homosexuality.

[CR37] Salway T, Juwono S, Klassen B, Ferlatte O, Ablona A, Pruden H, Morgan J, Kwag M, Card K, Knight R, Lachowsky NJ (2021). Experiences with sexual orientation and gender identity conversion therapy practices among sexual minority men in Canada, 2019–2020. PLoS ONE.

[CR38] Smith, A., Forsyth, K., Poon, C., Peled, M., Saewyc, E., & McCreary Centre Society. (2019). *Balance and connection in BC: The health and wellbeing of our youth*. McCreary Centre Society.

[CR39] Tan KKH, Wilson AB, Flett JAM, Stevenson BS, Veale JF (2022). Mental health of people of diverse genders and sexualities in Aotearoa/New Zealand: findings from the New Zealand Mental Health Monitor. Health Promotion Journal of Australia.

[CR40] Turban JL, King D, Reisner SL, Keuroghlian AS (2019). Psychological attempts to change a person’s gender identity from transgender to cisgender: Estimated prevalence across US States, 2015. American Journal of Public Health.

[CR41] Turban JL, Beckwith N, Reisner SL, Keuroghlian AS (2020). Association between recalled exposure to gender identity conversion efforts and psychological distress and suicide attempts among transgender adults. JAMA Psychiatry.

[CR42] VanderWaal CJ, Sedlacek D, Lane L (2017). The impact of family rejection or acceptance among LGBT+ Millennials in the Seventh-day Adventist Church. Social Work & Christianity.

[CR43] Veale, J. F., Tan, K. K. H., & Byrne, J. L. (2021). Gender identity change efforts faced by trans and nonbinary people in New Zealand: Associations with demographics, family rejection, internalized transphobia, and mental health. *Psychology of Sexual Orientation and Gender Diversity*. 10.1037/sgd0000537.

[CR44] Waterman, A. S. (1982). *Identity Development From Adolescence to Adulthood: An Extension of Theory and a Review of Research*. 18(3), 341–358.

